# Interventions to Reduce Opioid Use in Youth At-Risk and in Treatment for Substance Use Disorders: A Scoping Review

**DOI:** 10.1177/07067437221089810

**Published:** 2022-05-09

**Authors:** Stephanie A. Nairn, Marion Audet, Sherry H. Stewart, Lisa D. Hawke, Jason Y. Isaacs, Joanna Henderson, Rebecca Saah, Rod Knight, Danya Fast, Faria Khan, Alice Lam, Patricia Conrod

**Affiliations:** 1 Département de Psychiatrie, Faculté de Médecine, Université de Montréal, Montréal, Québec, H3T 1J4, Canada; 2 Centre de Recherche, CHU Ste-Justine, Montréal, Quebec, H3T 1C4, Canada; 3 Department of Sociology, 5620 McGill University, Montreal, Quebec, H3A 2T7, Canada; 4 Department of Psychiatry, 3688 Dalhousie University, Halifax, Nova Scotia, B3H 2E2, Canada; 5 Department of Psychology & Neuroscience, 3688 Dalhousie University, Halifax, Nova Scotia, Canada; 6 7978 Centre for Addiction and Mental Health, Toronto, Ontario, M6J 1H4, Canada; 7 Department of Psychiatry, University of Toronto, Toronto, Ontario, M5T 1R8, Canada; 8 Cumming School of Medicine, 70401 University of Calgary, Calgary, Alberta, T2N 4N1, Canada; 9 Department of Community Health Sciences, 70401 University of Calgary, Calgary, Alberta, T2N 4Z6, Canada; 10 British Columbia Centre on Substance Use, Vancouver, British Columbia, V6Z 2A9, Canada; 11 Research Centre du Chum, Montreal, Quebec, H2X 0C1, Canada

**Keywords:** youth, opioid use disorder, opioids, prescription opioids, intervention, trauma, biopsychosocial, gender, harm reduction

## Abstract

**Background:**

Youth and young adults have been significantly impacted by the opioid overdose and health crisis in North America. There is evidence of increasing morbidity and mortality due to opioids among those aged 15–29. Our review of key international reports indicates there are few youth-focused interventions and treatments for opioid use. Our scoping review sought to identify, characterize, and qualitatively evaluate the youth-specific clinical and pre-clinical interventions for opioid use among youth.

**Method:**

We searched MedLine and PsycInfo for articles that were published between 2013 and 2021. Previous reports published in 2015 and 2016 did not identify opioid-specific interventions for youth and we thus focused on the time period following the periods covered by these prior reports. We input three groups of relevant keywords in the aforementioned search engines. Specifically, articles were included if they targeted a youth population (ages 15–25), studied an intervention, and measured impacts on opioid use.

**Results:**

We identified 21 studies that examined the impacts of heterogeneous interventions on youth opioid consumption. The studies were classified inductively as psycho-social-educational, pharmacological, or combined pharmacological-psycho-social-educational. Most studies focused on treatment of opioid use disorder among youth, with few studies focused on early or experimental stages of opioid use. A larger proportion of studies focused heavily on male participants (i.e., male gender and/or sex). Very few studies involved and/or included youth in treatment/program development, with one study premised on previous research about sexual minority youth.

**Conclusions:**

Research on treatments and interventions for youth using or at-risk of opioids appears to be sparse. More youth involvement in research and program development is vital. The intersectional and multi-factorial nature of youth opioid use and the youth opioid crisis necessitates the development and evaluation of novel treatments that address youth-specific contexts and needs (i.e., those that address socio-economic, neurobiological, psychological, and environmental factors that promote opioid use among youth).

## Abrégé

**Contexte:**

Les jeunes et les jeunes adultes ont été significativement touchés par les surdoses d’opioïdes et la crise sanitaire en Amérique du Nord. Des données probantes indiquent une morbidité et une mortalité croissantes attribuables aux opioïdes dans le groupe des 15–29 ans. Notre revue des principales études internationales indique qu’il y a peu d’interventions axées sur les jeunes ni de traitements pour l’utilisation d’opioïdes. Notre revue de la portée cherchait à identifier, caractériser, et évaluer qualitativement les interventions précliniques et cliniques spécifiquement pour les jeunes qui utilisent des opioïdes.

**Méthode:**

Nous avons cherché dans MedLine et PsycInfo des articles publiés entre 2013 et 2021. Des rapports précédents publiés en 2015 et 2016 n’identifiaient pas d’interventions spécifiques des opioïdes pour les jeunes et nous avons donc mis l’accent sur la période suivant les périodes couvertes par ces rapports précédents. Nous saisissons trois groupes de mots-clés pertinents dans les moteurs de recherche susmentionnés. Spécifiquement, les articles ont été inclus s’ils ciblaient une population de jeunes (15–25 ans), étudiaient une intervention, et mesuraient les effets de l’utilisation d’opioïdes.

**Résultats:**

Nous avons identifié 21 études qui examinaient les effets des interventions hétérogènes sur la consommation d’opioïdes des jeunes. Les études ont été classées de façon inductive comme psycho-sociales-éducatives, pharmacologiques, ou une combinaison de pharmacologiques-psycho-sociales-éducatives. La plupart des études étaient axées sur le traitement du trouble d’utilisation d’opioïdes chez les jeunes, et peu d’entre elles se penchaient sur les stades précoces ou expérimentaux de l’utilisation d’opioïdes. Une proportion plus importante des études étaient lourdement centrée sur les participants masculins (c.-à-d., de sexe ou de genre masculin). Très peu d’études impliquaient ou incluaient des jeunes dans un développement de traitement/programme, et une étude reposait sur une recherche précédente sur la minorité sexuelle chez les jeunes.

**Conclusions:**

La recherche sur les traitements et interventions pour les jeunes utilisant ou à risque d’utiliser des opioïdes semble être clairsemée. Plus de participation des jeunes à la recherche et au développement de programmes est vitale La nature inter-sectionnelle et multifactorielle de l’utilisation d’opioïdes des jeunes et la crise des opioïdes chez les jeunes nécessitent le développement et l’évaluation de nouveaux traitements qui abordent les contextes et besoins spécifiques des jeunes (c.-à-d., ceux qui tiennent compte des facteurs socio-économiques, neurobiologiques, psychologiques et environnementaux qui favorisent l’utilisation d’opioïdes chez les jeunes).

## Introduction

The opioid overdose and health crises are complex and multi-factorial global challenges that impact several different populations.^1^ Morbidity and mortality due to opioid use has been documented in North America and in particular in the US; however, Canadian researchers, policy makers, and healthcare professionals also navigate the challenges of reducing the harms of opioid use, specifically among adolescent and emerging adult populations (ages 15–25 years). In 2017, there were 3,987 opioid-related deaths and 20% occurred among the age group 20 to 29 years.^2^ Over a recent 5-year period, younger adults had the fastest-growing rates of opioid poisonings.^3^ The COVID-19 pandemic exacerbated these trends and a recent provincial study showed the greatest increase in opioid-related deaths among the under 35 age group.^4^

Deaths occur not only among long-time substance users but among young people who have used them for the first time.^5^ Media coverage has focused on the circulation of potent narcotics like fentanyl and the roles of pharmaceutical manufacturers in perpetuating opioid-related morbidity and mortality.^6–8^ Despite a popular emphasis on these factors, the (youth) opioid crisis can be understood as an interactive combination of non-actions^9^ between opioid producers, medical care systems, government, and other crucial regulatory agencies and a biopsychosocial phenomenon that is inadequately addressed through an emphasis on drug supplies and pharmaceutical manufacturers’ practices.^10^

The aforementioned non-actions have been compounded by an absence of Canadian epidemiological data about non-medical opioid use (NMOU) among youth. Provincial surveys (e.g., the Ontario Student Drug Use and Health Survey and the Canadian Tobacco, Alcohol, and Drugs Survey) have served as key indicators for understanding NMOU among youth.^11,12^ The OSDUHS survey showed that ∼11% of grades 7 to 12 students reported using prescription opioids non-medically and 20% reported it was easy or very easy to acquire access to prescription opioids.^11^ These surveys suggest that NMOU rates have remained relatively stable since 2013, but the fact some youth are consuming prescription opioids non-medically is problematic for several reasons. Youth using heroin today are more likely to have initiated use via prescription opioids due to lower costs and greater accessibility^13^ and some qualitative studies have shown that youth view prescription opioids as less dangerous or more socially acceptable than other illicit drugs, although perceptions vary with different opioids.^14,15^

Youth perceptions of opioids are contextualized by neurobiological vulnerabilities, familial, peer, and socio-structural risk factors, and contexts. Youth are more susceptible to impulsivity,^16,17^ sensation seeking and disregarding negative consequences, and to potential cognitive impairment due to substance use that can continue into emerging adulthood.^18^ These vulnerabilities are contextualized by interactions with family members and peers, wherein diversion is thought to be ubiquitous.^19^ Indeed, 40% of Canadian students who consumed a prescription opioid were given them by a parent.^19^ A recent study showed that youth who live with a family member who have a prescription for opioids are at risk for overdose.^20^ Peer, romantic, and/or sexual partners’ opioid use are also risk factors for youth and youth perceptions of opioid use behaviours among their peer groups are predictive of actual opioid use.^21,22^ Homelessness, trauma, and socio-economic status (SES) are also key contextual risk factors, particularly for opioid use.^13^ Canadian youth with low SES are 2.4 times more likely to report using prescription pain relievers (e.g., opioids) than a youth of higher SES.^23^

Recent reports published in 2016^24^ and 2015^25^ noted there were few opioid-specific interventions for young people and most interventions have focused on alcohol, cannabis, and other illicit substances. The only programs that resulted in reduced use of prescription opioids for young people were the PROSPER framework and a personality-targeted intervention.^18,26,27^ A narrative review of pharmacological treatments for adolescents with opioid use disorder (OUD), discussed the efficacy and risks of various treatments.^28^ Across all three reviews, studies were predominantly from prior to 2013.

Due to continuing opioid-related harms to young people in Canada, it is crucial to know what is helpful for preventing and treating opioid use and to determine whether additional solutions have been identified since the publication of the reports. The objectives for this scoping review were to identify novel and promising interventions for youth who are currently using or at-risk for opioid use and to characterize and evaluate the evidence in favour of these treatments. Our review expands on the previous reports and review, as we utilized a systematic search strategy and focused broadly on psychological and pharmacological interventions. To date, there have been no systematic reviews of this kind in the domain.

## Methods

### Search Strategy

Our objective was to understand the characteristics of youth-focused novel treatments, the multiple ways they are studied, and their impacts on opioid use. We thus adopted a scoping methodology.^29^ We searched MedLine and PsycInfo for English studies that were published between 2013 and 2021 to capture interventions that were not included in the international reports.^24,25^ We input three groups of keywords that captured population, opioid use, and interventions.

The results were imported into EndNote. Titles and abstracts were screened by a research assistant (RA) and a PhD student independently. Full texts were subsequently screened by an RA and a PhD student. If there were discrepancies about whether studies should be included, both reviewers discussed the articles and consulted with the executive team to determine whether the articles should be included. The reference lists were screened to identify additional studies.

### Inclusion/Exclusion Criteria

The inclusion criteria were: (1) The study focused on a youth population (15–25 years of age as per the Surgeon General's report^24^) or youth sub-sample; (2) The study had to include an intervention; (3) The study had to measure impacts on opioid use or high-risk substance use (e.g., methamphetamine, cocaine, or polysubstance use). We excluded studies that focused on adults, that measured outcomes only for other substances (e.g., cannabis), and/or that referenced opioid use but not an intervention. Non-peer-reviewed studies were not included.

### Data Charting & Analysis

Data were extracted from the studies regarding the classification of drug use within samples, sample age range and/or mean, the study's focus (e.g., treatment of OUD), the study design (e.g., RCT), the methodologies used to gather data, opioid-specific outcomes, the sample size, the gender and/or sex of the participants, and the geographical location. Each variable was recorded in an excel spreadsheet by an RA and a PhD student.

## Results

**Figure 1.**
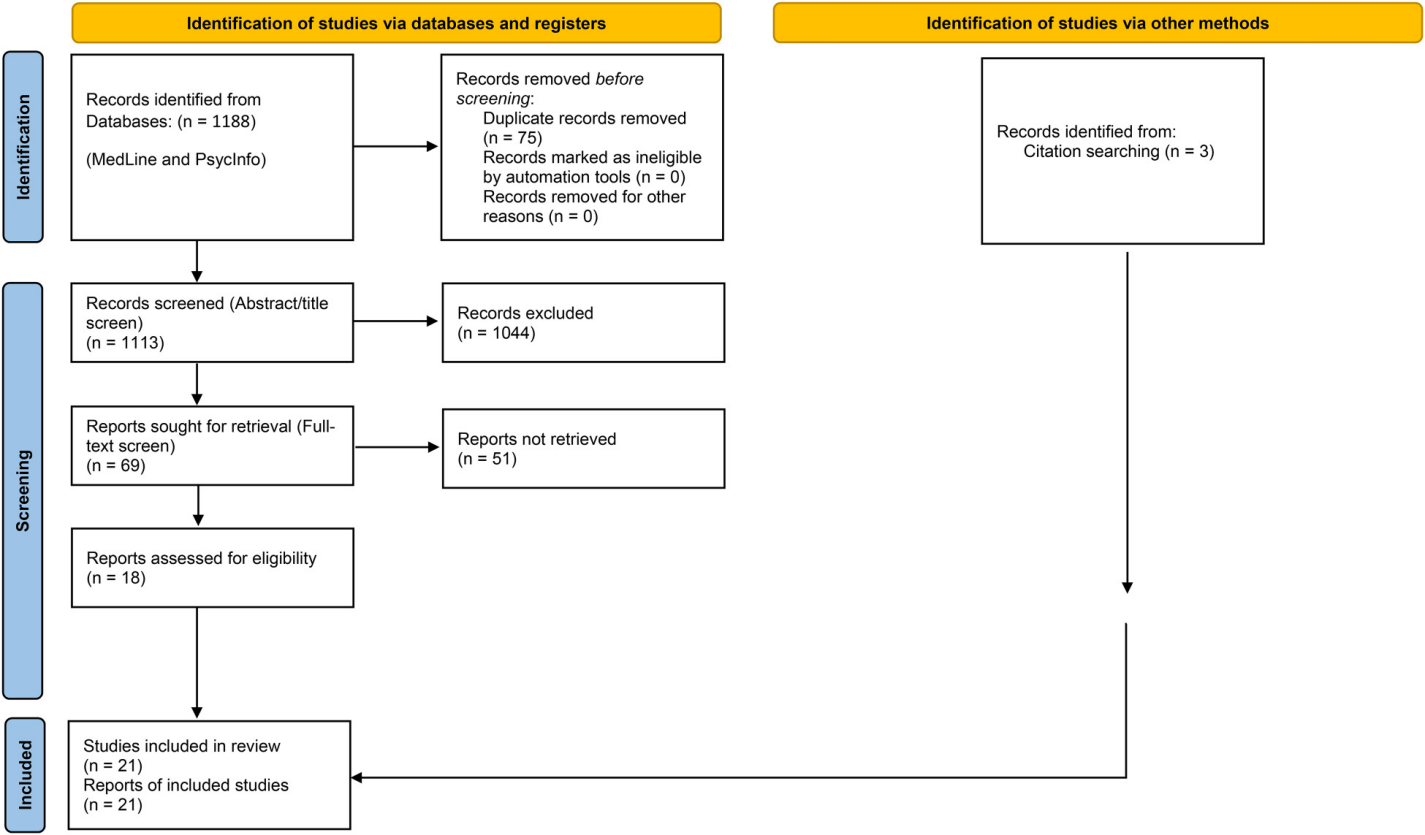
PRISMA flow chart for scoping review.

Combined searches of MedLine and PsycInfo resulted in 1,188 articles. Duplicates were removed (*n* = 75). The title and abstract scans resulted in the exclusion of 1,044 references. Sixty-nine full texts were screened for inclusion. Fifty-one articles were excluded after the full-text review and 18 were retained for qualitative synthesis. Three articles from citation searching were included, for a total of 21 studies (Figure 1).

The 21 studies were categorized inductively based on the types of studies. Two were classified as pharmacological, 8 were classified as psycho-social-educational, and 11 were classified as combinations of pharmacological-psycho-social-educational studies (Table 1).

**Table 1.** Interventions for Young Opioid Users Identified in the Literature.

*Pharmacological interventions*	
Cheng and Coplan^31^	OxyContin reformulation introduced on the market in 2010
Matson et al.^32^	Outpatient buprenorphine/naloxone clinic
*Psychosocial-educational interventions*	
Bertrand et al.^33^	10-weeks, abstinence-based residential treatment for drug addiction
Santis et al.^34^	Systematic Family Outreach Intervention (SFOI)
Danzer^35^	Multidimensional Family Therapy (MDFT)
Takis^36^	Psychodrama therapy
Hadland et al.^37^	Safe injection facility (SIF)
Chang et al.^37^	Computer-delivered and counsellor-guided, school-based early intervention
Schwinn et al.^30^	Web-delivered early intervention for sexual minority youth
Kanato and Leyatikul^39^	Compulsory, abstinence-based “drug camps”
*Combined interventions*	
Vo et al.^40^	Young adult alternative treatment program (YAAP)
Sigmon et al.^42^	Buprenorphine-naloxone stabilization and naltrexone therapy with behaviour therapy
Vo et al.^41^	Home-based delivery of extended-release naltrexone
Ruisenōr-Escudero et al.^45^	Opioid substitution therapy clinic
Romero-Gonzalez et al.^46^	Buprenorphine-naloxone medication with group cognitive behavioural therapy
Ghaddar et al.^47^	Opioid agonist therapy program
Ghaddar et al.^48^	Opiate agonist treatment
Gonzalez et al.^43^	Buprenorphine-Naloxone therapy with or without memantine
Jhugroo et al.^49^	Sustained-release naltrexone implant
Law et al.^44^	Detoxification and opioid substitution therapy
Schuman-Olivier et al.^50^	Collaborative care buprenorphine treatment program

### Sample Characteristics

Ten studies recruited youth from the US, 2 from Canada, 2 from Lebanon, and 7 studies were from other countries (see Table 2). All study samples had either a mean age between 15 and 25 years (*n* = 17) or highlighted youth-specific findings, despite a slightly higher average age among the total sample (*n* = 4). A total of 17 of the 21 studies focused disproportionately on male participants (e.g., a sample that incorporated over 50% male participants) or exclusively on the treatment of male participants. One study purposively recruited a sample of “sexual minority youth”^30^ (i.e., queer, gender fluid, and/or “other” genders) alongside women and men. Eight studies reported gender as binary (i.e., male or female). Two studies did not report sample sex or gender, 6 reported on “male” and “female” participants or “men” without referring to whether the status was regarded as sex or gender, and three studies reported “sex.”

**Table 2.** Characteristics of Included Studies.

Intervention	Location	Sample size	Age	Gender/sex	Type of drug use	Study design	Data collection methods
*Pharmacological interventions*							
OxyContin reformulation	USA	∼600,000	12 +	N/A	Non-medical or extra-medical OxyContin use	Retrospective cross-sectional study	Computer-assisted self-interviews
Medication-Assisted Treatment for Addiction (MATA) clinic	USA	103	Mean of 19.2	50.5% male	Opioid dependence (prescription and/or heroin)	Retrospective chart review	Patient charts (which included results of urine tests)
*Psychosocial-educational interventions*							
10-weeks, abstinence-based residential treatment for drug addiction	Canada	102	14–18	60% male	Addiction to drugs (including opioids)	Longitudinal study in a natural clinical setting	Questionnaires and patient charts
Systematic Family Outreach Intervention (SFOI)	Chile	67 in EG and 71 in CG	<25 (mean of 18 in EG and 19 in CG)	69% male in EG, 65% in CG	Addiction to marijuana, cocaine, alcohol, and “other” drugs	Quasi-experimental study with comparison condition	Questionnaires
Multidimensional Family Therapy (MDFT)	USA	1	17	100% male	Use of alcohol and over-the-counter drugs	Case study	Interviews
Psychodrama therapy	Greece	1	20	100% male	Addiction to heroin and cannabis	Case study	Observations
Safe injection facility	Canada	414	14–26	66% male	Injecting drug use (heroin, cocaine, crystal meth)	Prospective cohort study	Questionnaires
Computer-delivered and counselor-guided school-based early intervention	Taiwan	84 (43 in IG)	Mean of 17	67% male in IG and 75% male in CG	Experimenting with amphetamines, ketamine, marijuana, ecstasy	Randomized controlled trial	Questionnaires and urine testing
Web-delivered early intervention for sexual minority youth	USA	236 (119 in IG)	15–16	32% male and 18% queer/fluid/other in IG and 33% male and 14.5% queer/fluid/other in CG	Use of alcohol, cigarettes, marijuana, inhalants, club drugs, steroids, cocaine, methamphetamine, prescription drugs, and heroin	Randomized controlled trial	Questionnaires
Compulsory “drug camps”	Thailand	2679 (24 opioid users)	Mean of 24.8	92.6% male	Any drug use	Longitudinal study	Questionnaires and observations
*Combined interventions*							
Young Adult Alternative Treatment Program (YAAP)	USA	56	Mean of 23.1	70% male	Addiction to opioids	Naturalistic study using retrospective chart review	Patient charts (which included results of urine tests)
Buprenorphine-naloxone stabilization and naltrexone therapy with behavioral	USA	70	Mean of 27.5	69% male	Dependence on prescription opioids	Randomized double-blind trial	Questionnaires, urine testing and patient charts
Home-based delivery of extended-release naltrexone	USA	14 ( + 21 from previous cohort comparison)	17–25	64% male	Addiction to opioids	Naturalistic case series with historical comparison group	Patient charts
Opioid substitution therapy clinic	Afghanistan	83	18–24	100% male	Dependence on heroin	Cross-sectional assessments	Questionnaires
Buprenorphine-naloxone medication with group cognitive behavioural therapy	USA	63 prescription drug users and 17 heroin users	18–25	66% male	Dependence on opioids (prescription vs. heroin)	Prospective cohort study	Questionnaires, urine testing and patient charts
Opioid agonist therapy program	Lebanon	81	Mean of 29	100% male	Opioid use disorder	Cross-sectional study	Interviews
Opiate agonist treatment	Lebanon	86 at entry and 3 months, 38 at 12 months	18–66 (median of 28)	100% male	Opioid dependence	Pre-post design	Interviews, questionnaires, and urine testing
Buprenorphine-Naloxone treatment	USA	87 at randomization, 80 in intent-to-treat sample	18–25 (mean of 22)	66% male	Opioid dependence	Randomized, double-blind, placebo-controlled trial	Questionnaires, urine testing, observations, and laboratory tests
Sustained-release naltrexone implant	Australia	24	19–28 (mean of 24)	NA	Buprenorphine dependence	Observational case series	Drug testing
Opioid substitution therapy	UK	80	Mean of 23 (for methadone group) and mean of 23.2 (for buprenorphine group)	NA	Opioid dependence	Pragmatic randomized controlled trial	Urine testing, questionnaires (e.g., craving scales)
Collaborative care buprenorphine treatment program	USA	294 (70 of which were emerging adults)	Mean of 23.1	64.3% male	Polysubstance users	Retrospective chart review	Urine testing, comprehensive care assessments

The classification of opioid and other drug use among the samples was *heterogeneous.* In the pharmacological studies, opioid use was defined as non-medical or extra-medical OxyContin use and opioid dependence. In the psycho-social-educational interventions, youth were classified as “addicted to opioids” and “other drugs,” youth using drugs (e.g., cocaine, methamphetamine, prescription drugs, and heroin), injecting drugs (e.g., heroin, cocaine, and crystal meth), and experimenting with amphetamines. Youth were primarily engaged in prescription opioid (PO) use and/or experienced OUD, or were “experimenting.” In the combined interventions, youth were “addicted to opioids,” “dependent on prescription opioids,” “dependent on heroin,” buprenorphine dependent, seeking buprenorphine treatment, or had OUD.

### Characteristics of Study Designs

### Pharmacological Interventions

One pharmacological study was a retrospective, cross-sectional study in the US and assessed the incidence of non-medical opioid use before and after the introduction of abuse-deterrent OxyContin in 2010 with an oversampling of youth 12 to 17 years.^31^ The additional pharmacological study was a retrospective chart review of adolescents who sought treatment at a Medication-Assisted Treatment for Addiction clinic (MATA). The clinic provided outpatient buprenorphine/naloxone to youth (ages 14–25 years) and helped “manage” other medical and mental health conditions. All participants were prescription opioid-dependent or combined heroin/prescription-opioid dependent.^32^

### Psycho-Social-Educational Interventions

Youth in the psycho-social-educational studies were younger than youth in the pharmacological/combined interventions and there was a heterogeneity of study designs and methods to impact opioid use and other substance use.

One study was longitudinal and assessed a residential treatment program.^33^ A total of 65% of the sample used both alcohol and other drugs, including heroin, opiate, analgesics, “narcotics,” methamphetamine/speed, and cocaine. Another study examined a Systematic Family Outreach Intervention (SFOI)^34^ and incorporated a quasi-experimental design and a comparator group (i.e., traditional outreach work). Participants were recruited from “communes” with low-income populations, high rates of substance use and drug trafficking. Approximately 75% of the sample were polysubstance users including alcohol, marijuana, cocaine, and “other” drugs.

Two psycho-therapeutic case studies involved Multidimensional Family Therapy (MDFT)^35^ and psychodrama therapy.^36^ MDFT involved counselling sessions with the participant and his family members. They were living in a low-income household and the participant had a history of alcohol use and an overdose on over-the-counter substances. There was weekly drug testing. The psychodrama case study involved a participant using heroin and cannabis and included weekly group sessions and one-on-one counselling. His family dynamic was described as “hostile” and “dysfunctional.”

The At-Risk Youth Study (ARYS) was a prospective cohort design and assessed youth use of a Safe Injection Facility (SIF).^37^ Participants were street-involved and injected heroin and crystal meth. The population was described as at-risk for morbidity and mortality.

Additional psycho-social-educational studies were computer/web-delivered interventions. The computer-delivered and counsellor-guided intervention^38^ was an RCT and completed in schools with youth experimenting with amphetamines, marijuana, ketamine, and ecstasy; 50% reported substance use (SU) among their peers. The web-delivered intervention was also an RCT and targeted sexual-minority youth using alcohol, cigarettes, marijuana, and other drugs (cocaine, methamphetamines, prescription drugs, and heroin). The self-report questionnaires implemented at pre-, post-, and 3-month follow-up recorded information on peer SU.^30^

A longitudinal study from Thailand examined compulsory “drug camps” with youth using drugs.^39^ The camps were standardized, 9-day rehabilitation programs involving recreational and boot camp activities, drug resistance education, “drug abuse rehabilitation procedures” and occupational training. Drug use and demographic questionnaires were completed by participants. Participant observation procedures were also used. Less than 1% of camp attendees reported using opioids prior to camp and most attendees were amphetamine, alcohol, and polydrug users (31%).

### Combined Pharmacological-Psycho-Social-Educational Interventions

The combined studies analyzed variations of Opioid Agonist Therapy (OAT) alongside several psycho-social-educational modalities. Seven of the 11 studies assessed combinations of buprenorphine treatments and other pharmacological and psychosocial adjuncts. There was a heterogeneity of study designs and all studies involved outpatient treatment(s), with one study providing the option to remain in inpatient treatment, before transitioning to outpatient treatment. The combined studies assessed primarily males/men, ranging from 64% to 100% male representation.

A naturalistic and retrospective chart review of 56 patients explored the Young Adult Alternative Treatment Program (YAAP) that provided youth with the option of taking buprenorphine or extended-release naltrexone.^40^ Participants had access to counselling, mental health therapy, and psychiatric treatment. Participants had an OUD and were polysubstance users; 89% had a psychiatric diagnosis and 82% injected heroin.

An additional naturalistic study compared home-based delivery of extended-release naltrexone (XR-NTX) by a nurse practitioner with those who had received clinic-based delivery of the same medication in the previous year.^41^ The community-based program involved residential detoxification and a 7-day buprenorphine taper for adolescents and young adults with opioid addiction to prescription opioids and heroin; 64% of the participants injected heroin. Youth were invited to continue inpatient treatment before transitioning to outpatient treatment. The youth had the option of receiving counselling at home.

An outpatient double-blinded RCT assessed the effects of a combination of buprenorphine hydrochloride and naloxone hydrochloride dihydrate via 1-, 2-, or 4-week tapers. Participants were dependent on prescription opioids, 41% had used the intravenous route at least once and 89% were polysubstance users. Participants were provided with individual behavioural therapy.^42^ The sample had high rates of employment, education, and low rates of medical and psychiatric problems.

An additional 13-week double-blinded RCT compared treatment of opioid-dependent young adults with buprenorphine-naloxone or placebo with 15 or 30 mg of memantine. All participants were offered weekly group cognitive-behavioural therapy.^43^

An additional RCT compared the efficacy of lofexidine/methadone and buprenorphine/naloxone during opiate withdrawal following opiate stabilization on methadone and buprenorphine/naloxone among opioid-dependent users.^44^ The study included medical/general health questionnaires, physical examinations, and blood tests. The participants were stabilized daily, followed by detoxification. Participants were then treated with either lofexidine/methadone or buprenorphine/naloxone. Vouchers for local grocery stores were provided and this was contingent on opioid negative urine, attendance, and completion of questionnaires.

Two additional studies incorporated prospective cohort designs and assessed variations of OAT. One study included injecting male heroin users and combined methadone with psychosocial therapy, education, recreational, and medical services.^45^ Another study assessed buprenorphine-naloxone doses over an 8-week period, after opioid-dependent youth were abstinent from either heroin or prescription opioids.^46^ Youth could attend weekly group cognitive behavioural sessions. The participants had employment problems and were likely to be depressed.

An additional cross-sectional study of young men with OUD attending an OAT program^47^ integrated psychiatric and psychological care, as well as provided access to a nurse and social worker. A longitudinal study by the same researchers and a different cohort examined the effects of OAT and psychosocial support on opioid-dependent males at the first outpatient community-based treatment centre in Lebanon.^48^

An observational case series of 24 opiate-dependent participants evaluated the effects of a double naltrexone implant treatment. Patients travelled from Mauritius to Australia to undergo surgery and follow-ups were done in Mauritius. Patients were provided with 20 min of brief counselling.^49^

A retrospective chart review examined opioid-dependent emerging adults’ experiences of a collaborative care buprenorphine treatment program.^50^ The program consisted of brief detoxification and buprenorphine induction. The participants were required to attend weekly one-on-one or group psychosocial treatment sessions. A total of 81.4% of the participants reported lifetime use of heroin.

## Outcomes of the Studies

### Pharmacological Studies

The study examining the incidence of non-medical OxyContin use before and after the abuse-deterrent formulation was released in 2010, showed that there was a 50% reduction in new incidences of OxyContin use between 2010 and 2012 for youth aged 12 to 21 years. However, the same trend of reduced incidence was not observed for youth's NMOU of other prescription opioids (see Table 3 for study outcomes).^31^

**Table 3.** Outcomes of Included Studies.

Intervention	Outcomes	Rigor scores (out of 16)
*Pharmacological interventions*		* *
OxyContin reformulation	Reduced incidences of non-medical OxyContin use after introduction of abuse-deterrent OxyContin formulation, with a 50% reduction in new cases for youth 12 to 21 between 2010 and 2012, but not for youth's non-medical use of other POs	11—strong
Medication-Assisted Treatment for Addiction (MATA) clinic	Opioid-dependent adolescents and young adults had high rates of compliance with medication and of opioid abstinence while engaged in treatment (85.2% and 86.6% respectively). However, after 60 days, 45% of patients were retained, with retention rates dropping to 9% at one year. Female sex was reported to be significantly associated with higher retention rates.	10—moderate
*Psycho-social-educational interventions*		* *
10-weeks, abstinence-based residential treatment for drug addiction	More severe drug problems at entry were associated with older age, higher levels of mental health problems, better therapeutic alliance, and faster decline in rates of use during treatment. Number of post-treatment sessions attended was positively related to reduction in drug use.	6—moderate
Systematic Family Outreach Intervention (SFOI)	Intervention group obtained better scores on drug severity scale, but reported worse family conflict (hypothesized to be due to increased family awareness of youth's problematic drug use)	9—moderate
Multidimensional Family Therapy (MDFT)	Improved grades by end of treatment, increased self-reported self-esteem and reduced drug use and conduct problems. Relapse episode after relocation to new city.	2—modest
Psychodrama therapy	Suggests patient has a more “consolidated sense of self, a new role, oriented towards development and self-growth and a desire to leave behind old roles” (Takis, 2018). No report of complete cessation of heroin use, but expressed intention/desire to be free of the “chains” of heroin use	2—modest
Safe injection facility	42% of sample used the SIF at least once: 71% of which had recently used it at enrollment and 29% had not but used it at least once during follow-up. More than half of SIF users reported using it every week and performing 25% of injections at the site. Users of the SIF were more likely to live or spend time near the SIF, to inject in public, or to inject heroin or crystal methamphetamine daily.	6—moderate
Computer-delivered and counsellor-guided school-based early intervention	There were no significant improvements at end of main intervention, but changes became significant after booster sessions: no positive drug test for EG after booster versus to 2 positive tests in CG. Statistically significant improvements in stress management, drug refusal skills, self-efficacy, and decrease in identification of the “pros” (i.e., the perceived individual benefits) of drug use over time according to self-reports on questionnaires for EG.	8—moderate
Web-delivered early intervention for sexual minority youth	At 3 months follow-up, less stress, less drug use among peers, and less past 30-day other drug use (i.e., other than alcohol, cigarettes, or marijuana) higher coping and problem-solving skills, and better drug use refusal abilities for IG versus CG.	7—moderate
Compulsory “drug camps”	Drug use was reported to drop from 70.1% prior to camps to 45.8% after, and 41.1% a year after the end of camp. However, data also showed increases in the number of poly-drug users after spending time at the camps in those 14 years and older, and drug use increased after the camps for most drug types. Opioid use increased from 0.9% before camps, to 2.8% 3 months after camps, and 2.7% at 1 year.	6—moderate
*Combined interventions*		* *
Young Adult Alternative Treatment program (YAAP)	Retention fell from 65% at 12 weeks to 40% at 24 weeks, while rates of negative opioid urine tests went from 50% to 39% over the same period. No significant differences in clinical baseline characteristics between the older (24 +) and younger (< 24) age group. No significant differences in treatment outcomes (retention rates, attendance, rates of opioid-negative urine tests) according to age or medication (BUP or XR-NTX) received. Males performed better than females overall.	9—moderate
Buprenorphine-naloxone stabilization and naltrexone therapy with behavioural	4-week taper produced superior outcomes over briefer durations, with 50% of retained participants remaining abstinent (negative weekly urine testing) and receiving naltrexone at the end of the study versus 17% and 21% for the 2- and 1-week tapers, respectively.	14—strong
Home-based delivery of extended-release naltrexone	After 4 months in outpatient treatment, retention rates for the study group were 64% and 50% of the participants took all 5 doses versus 19% retention and 9% taking all 5 doses for CG. Both groups attended a similar number of counselling sessions and discontinuation of opioid use was observed in most patients. Within-group rates and between-group differences in opioid discontinuation were not reported.	6—moderate
Opioid substitution therapy clinic	End line group was found to be significantly healthier, have improved family contact, have a source of income and report fewer illegal activities and less heroin use than baseline group. Those who remained in treatment tended to be older, single, report fewer psychotic symptoms, and had daily contact with family in the past month. Being 18 to 24 years olds was significantly associated with increased loss to follow-up.	7—moderate
Buprenorphine-naloxone medication with group cognitive behavioural therapy	Similar rates of retention (99% for PO vs. 96% for heroin), reduction of craving symptoms, and reduction of withdrawal symptoms were reported for both groups. Reduction in opioid use was greater for PO users than heroin users, and only PO users had improved depression scores at 8 weeks.	7—moderate
Opioid agonist therapy program	The percentage of injecting drug users went from 58% at baseline to 12% after therapy. Respondents were split into two groups: those with financial and/or logistical barriers to accessing treatment versus those without. Both groups expressed concerns over dependence on buprenorphine. The “no barriers” group reported more improvements in physical and mental health, reductions of drug cravings, abstinence from other drugs, social functioning, relationships, self-image, and confidence than the “barriers” group. The “barriers” group reported more concerns over side-effects of treatment, costs of treatment, and adherence to treatment protocol compared to the “no barriers” group.	5—modest
Opiate agonist treatment	Treatment showed positive effects on drug use outcomes, with significant reductions in rates of opioid dependence and opioid use disorder diagnoses for heroin, cocaine, and cannabis use, as well as overdose incidence, at 3 and 12 months. At 3 months, a significant reduction was also observed on the score of depression and sharing needles, and significantly more people were employed. Reductions in arrests and anxiety scores as well as increases in quality-of-life scores were significant at both 3 and 12 months.	8—moderate
Buprenorphine-Naloxone therapy with or without memantine	Medication compliance was over 82% with no significant differences between groups. Retention rates were also similar across groups, dropping from 85% at week 8 to 50% at week 10, and 25% at week 13. Compared to the placebo and memantine 15 mg group, the memantine 30 mg group showed a significantly greater reduction in opioid use over time, and was significantly more likely to remain abstinent after rapid buprenorphine discontinuation at week 9. Opioid cravings and withdrawal symptoms for this group were also reduced significantly more than the other two groups, but only after buprenorphine discontinuation. The difference in withdrawal symptoms was non-significant for the last 2 weeks of treatment.	12—strong
Sustained-release naltrexone implant	Abstinence was 100% at 6 months, 83% at 1 year, 46% at 1.5 years, and 42% at 2.5 years. 79% of participants said they tried to inject buprenorphine or heroin in the first months post-surgery to override the naltrexone implant blockade, but were all unsuccessful. Following relapse, two patients returned from Mauritius to Australia for re-treatment, and another returned for re-treatment despite having remained abstinent. Others also expressed the desire to be re-treated, but were not able to make the trip to Australia.	5—modest
Opioid substitution therapy	A number of completed detoxifications were similar across both groups, with 23 completions in the methadone/lofexidine group and 21 in the buprenorphine/naloxone group. The methadone/lofexidine group had significantly worse subjective withdrawal symptoms and higher heart rates than the buprenorphine/naloxone group. There were no significant differences between groups in overall reported cravings, negative urine samples, and systolic or diastolic blood pressure.	13—strong
Collaborative care buprenorphine treatment program	Retention rates were significantly lower for emerging adults compared to older adults at 3 and 12 months.	10—moderate

The MATA study indicated that missing urine drug screening rates were low and buprenorphine-naloxone compliance rates and opioid abstinence rates were high while youth were engaged with the clinic. Retention was a major barrier to the success of treatment; 75% returned for a second visit, after one visit, at 2 months, 45% of patients were retained and at 1 year, 9% of patients were active in the program. The authors noted that the female sex was associated with higher retention.^32^

### Psycho-Social-Educational Interventions

Results of the 10-week residential treatment^33^ demonstrated that youth with the most severe drug problems at entry were usually older and experienced the fastest decline in SU during treatment. Higher levels of mental health problems and better therapeutic alliance were associated with higher severity of SU problems at entry. The number of post-treatment sessions attended was positively associated with reductions in SU. Past 30-day heroin, opiate, narcotic, and analgesic drug use were reported and showed a reduction between entry and the 3-month follow-up, with an observed increase at the 6-month follow-up.

The SFOI study^34^ demonstrated the intervention group obtained better scores on the addiction severity scale compared to control (traditional outreach work) but reported worse family conflict.

The MDFT case study reported improved grades, increased self-esteem, reduced drug use, and conduct problems, and a relapse episode when he relocated to a new city.^35^ The psychodrama case study reported a more “consolidated sense of self, a new role oriented towards development, self-growth and a desire to leave behind old roles” (p. 341).^36^ The report also mentioned the participant's desire to be free of the “chains” of heroin use.

A total of 42% of the participants in the SIF^37^ study used the facility at least once, and 71% of participants used it around the time of enrollment in the study. More than half of SIF users used it every week and performed 25% of their injections at the site. SIF use was associated with daily heroin injection, daily cocaine injection, and having visited a crack house or shooting gallery.

The computer-delivered intervention^38^ resulted in zero positive urine tests (i.e., for use of amphetamines, ecstasy, ketamine, and cannabis) only after the booster sessions, compared to 2 positive urine tests in the control group. Inferential statistics to determine statistical significance were not provided due to a large number of cell counts at zero. The experimental group showed statistically significant improvements in comparison to the control group in stress management, drug refusal skills, self-efficacy, and a decrease in the identification of the ‘pros’ of SU after the boosters.

The web-delivered intervention for sexual minority youth^30^ resulted in less stress, less SU among peers, less past 30-day other SU (i.e., use of heroin, methamphetamines, inhalant, club drug, steroid, prescription drugs, and cocaine), higher coping and problem-solving skills, and better drug refusal abilities than the control groups.

The results of the compulsory drug camps^39^ were changes in current use from 70.1% before the camps, to 45.8% after the camps, and 41.1% 1 year after the camps. The decrease was accounted for via significant reductions in amphetamine-type stimulant use only. The authors noted that drug use *increased* for most drug types, including opioids, after the camps (i.e., for opioids from 0.9% before, to 2.8% 3 months after and 2.7% after 1 year). They noted that data from a community study (not cited) showed that many camp attendees became polysubstance users after the camps.

### Combined Pharmacological-Psycho-Social-Educational Interventions

The YAAP study^40^ reported reduced retention rates over time from 65% at 12 weeks to 45% at 24 weeks. No significant differences in baseline characteristics were observed between older (over 24 years) and younger (under 24 years) youth or in treatment outcomes (i.e., retention rates, attendance, rates of opioid-negative urine tests) according to age or medication (BUP or XR-NTX) received. More males than females completed the program.

The 4-week taper of buprenorphine-naloxone produced superior outcomes when compared to briefer tapers in the combined buprenorphine-naloxone, naltrexone, and behavioural therapy study.^42^ Indeed, 50% of retained participants in the 4-week taper remained abstinent from opioids after 12 weeks in comparison to 17% and 21% for the 2- and 1-week tapers, respectively. A total of 50% of 4-week taper participants were also receiving naltrexone at the end of the study, compared to 17% for the 2-week taper and 25% for the 1-week taper group. The difference in naltrexone ingestion between the 4-week group and the 2-week group was significant.

After 4 months in outpatient treatment, the home-delivery of ER naltrexone resulted in 64% retention, in comparison to 19% in the control. A total of 50% of the participants in the experimental group took all 5 doses, in comparison to 9% in the control.^41^ Both groups attended a similar number of counselling sessions. Seven of the 9 patients in the experimental group were abstinent from opioids at 4 months. Controls were not assessed for opioid abstinence precluding direct comparisons.

At end-line, male youth enrolled in the OST study were shown to be significantly healthier, have improved family contact and a source of income, report fewer illegal activities and have less heroin use compared to baseline participants.^45^ They were also more likely to be single, older, report fewer psychotic symptoms, older age at first heroin use, and in the past month, have greater daily contact with family.

The study of buprenorphine-naloxone medication with group cognitive behavioural therapy for heroin users in comparison to PO users, showed similar rates of retention for both groups.^46^ A statistically significant difference was observed in weekly urine toxicologies; 99% of PO users were negative in comparison to 96% of heroin users over 8 weeks. Reductions in craving and withdrawal symptoms were similar in both groups. Reduction in opioid use was greater for PO users than heroin users and only PO users had improved depression scores at 8 weeks.

The study that assessed young men attending OAT defined participants as those with financial barriers and those with no financial barriers to access.^47^ Men with no barriers reported more social, physical, and mental benefits of participation including reduced cravings, improved social standing, relationships, and mental health. Both groups worried about dependence on OAT and participants with barriers expressed concerns about treatment benefits. The therapy reduced injecting behaviour from 58% to 12% after the therapy in both groups.

The longitudinal study of the Lebanese OAT clinic showed reduced rates of opioid dependence, overdose incidences at 3 and 12 months, and OUD diagnoses for heroin, cocaine, and cannabis users. At 3 months, a significant reduction was observed in depression scores, needle sharing, and more participants were employed. The reductions in arrests, anxiety scores, and increases in quality-of-life were significant at 3- and 12-month follow-ups.^48^

In the double-blinded RCT comparing buprenorphine-naloxone with either placebo, memantine 15 or 30 mg, 82% of participants were compliant with medication. Retention rates were similar in both groups; 85% were retained at week 8, 50% at week 10, and 25% at week 13. The 30 mg group showed a greater reduction in opioid use over time and were more likely to remain abstinent after rapid buprenorphine discontinuation. The 15 mg group did significantly worse than the other 2 groups with regards to reductions in opioid use at weeks 5 and 8.^43^

Analysis of the naltrexone implant^49^ showed that abstinence from opioid use was 100% at 6 months, 83% at 1 year, 46% at 1.5 years, and 42% at 2.5 years. A total of 79% of participants tried to inject buprenorphine or heroin post-surgery, but all were unsuccessful. Two patients returned to Australia for re-treatment, and another returned for re-treatment despite having remained abstinent. Other participants expressed desires to be re-treated but could not make the trip to Australia.

The collaborative care buprenorphine treatment program demonstrated that emerging adults had lower retention rates than adults at 3 and 12 months.^50^

The RCT comparing buprenorphine/naloxone and methadone/lofexidine^44^ reported that 44 out of 80 youth successfully completed detoxification. There were no significant differences between the groups who completed detoxification and no significant differences reported in overall reported craving, the proportion of negative opioid urine samples, or systolic or diastolic blood pressure. The methadone/lofexidine participants had significantly worse levels of subjective withdrawal symptoms and higher heart rates than the buprenorphine/naloxone group.

**Table 4.** Synthesis of the Results of the Literature Search.

Location	Sample size	Age range	Gender/sex of participants	Type of drug use	Intervention type	Study design	Data collection method
*Pharmacological*							
North America	103–∼600,000	12 +	N/A–50.5% male	Non-medical or extra-medical opioid use and opioid dependence	Abuse-deterrence and medication-assisted treatment	Retrospective cross-sectional study and retrospective chart review	Computer-assisted self-interviews and patient chart review
*Psychosocial-educational interventions*							
North America, South America, Europe, Asia	1–2,679	11–67	32–100% male	Drug use, over-the-counter drug abuse, injection drug use, and drug addiction	Residential abstinence-based treatment, family-oriented therapy, psychodrama therapy, harm reduction, school-based and computer-assisted early interventions, compulsory “drug camps”	Randomized controlled trials, case series, prospective cohort study, longitudinal studies, quasi-experimental study with the comparison group	Questionnaires, interviews observations, and drug testing
*Combined*							
North America, Central Asia, Middle East, Europe, Australia	14–294	17 +	64–100% male	Opioid use, opioid dependence, and opioid addiction	Medication-assisted treatment and psychosocial treatment (group and one-on-one), behavioural therapy or counselling, contingency management	Randomized controlled trials, retrospective chart reviews, cross-sectional studies, case series, prospective cohort study, pre-post study	Questionnaires, interviews, drug and HIV testing, retrospective chart review, comprehensive care assessments, observations, laboratory tests

## Discussion

Of the total number of studies, we found very few targeted adolescents and young adults (Table 4). The studies that targeted youth were heterogeneous pharmacological, psycho-social-educational, and combined interventions that varied with regards to the samples, the types of interventions and the outcomes reported and achieved.

A notable finding was that almost all of the youth-focused interventions were not tailored a priori to specific youth populations. The intervention for sexual minority youth^30^ was the only study that was both tailored a priori through content and practice scenarios to the needs of a potentially marginalized youth population and involved a substantial proportion of (50%) women, queer, and gender-fluid participants. The majority of other studies enrolled over 60% of male participants. This is problematic as the evidence demonstrates young women are uniquely vulnerable to NMOU. They are more likely than men to be prescribed an opioid and to consume opioids for pain relief. Trans and other gender diverse youth are vulnerable to opioid use and were not included in the studies. Future studies could purposively recruit women, gender diverse youth, and integrate youth-tailored materials in interventions to improve treatment relevance and efficacy.

The studies we found focused on the treatment of youth who are already experiencing dependence. This could be due to the limitations of our search terms or indicate a lack of preventive programs for youth in the experimental stages of OU. The computer-delivered intervention in schools was an exception because it focused on youth “experimenting” with substances.^38^ Given the frequency of polysubstance use and the improvements observed in stress management, refusal skills, and self-efficacy, the results were nonetheless encouraging. Healthcare professionals should develop and evaluate preventative programs aimed at youth experiencing problems as they explore opioid use.

The majority of studies focused on treatment retention outcomes. Three studies noted older youth had higher retention and faster declines in rates of opioid use during (combined) treatments.^33,45,50^ The YAAP study found no differences between older and younger participants in retention or reductions in opioid use.^40^ Reduced retention over time in several studies may indicate that retention is a barrier to experimental treatment success. The MATA study noted that female sex was associated with higher retention, but this was not reflected on by Matson et al.^32^ There is a need to develop an in-depth understanding of factors that promote retention and of how to engage youth throughout the duration of the programs.

The guiding principle of several studies was abstinence from opioids or reductions in use. This may be a deterrent to youth enrollment, as abstinence all the time is often unrealistic. The use of abstinence-based “camps” in Thailand^39^ is an example of the abysmal failures of compulsory and abstinence-based approaches for young people. Within a paradigm of harm reduction, the goals of the interventions could include continued safer use to prevent iatrogenic effects of treatments.

The youth opioid overdose and health crises are multi-factorial and we found that consideration of socio-structural factors were not explicitly integrated into the study methodologies, yet were illustrated in more detail in the results. The psychodrama^36^ case study referred to the participant’s “hostile” family dynamic, family members were counselled in the MDFT^35^ and involved in the SFOI.^34^ The involvement of family requires careful consideration as youth reported worsened family conflict after the SFOI and improved family relations after the MDFT. With regards to peers and partners, the intervention for sexual minority youth^30^ was the only study that reported reduced peer substance use. Future studies could focus on integrating peers and romantic partners in treatments.

SES was also addressed in the results of some of the studies but was not discussed explicitly a priori*.* The MDFT,^35^ SIF,^37^ and SFOI^34^ studies recruited youth with low SES and/or who were street-entrenched. Financial factors were mentioned in the results of the 2 studies. Young men in OAT with no financial barriers experienced more benefits than men with financial barriers, suggesting this factor can negatively influence the ability to benefit from OAT.^47^ Prescription opioid users in the Sigmon et al.^42^ study had high rates of education and employment, which suggests OU and related harms transcends SES and confirms opioid users can come from a variety of backgrounds. One study^44^ surmised that contingency management promoted high treatment attendance rates and healthcare professionals could integrate novel approaches like contingency management in treatments of OUD for youth and consider their economic circumstances in the design of interventions.

It was promising to find studies that provided choices for youth within treatments.^40,41,45–47^ Several studies offered *options* to participate in counselling, but most studies did not report on whether/how often these options were taken up. It was impossible to discern how they may have differentially impacted the outcomes and going forward researchers should, in collaboration with youth, characterize the impacts of the various combined treatments on study outcomes.

### Limitations

We did not develop a search around individual-level risk factors, which could be a strategy to identify additional interventions. Hopelessness is linked with OU among high school and college students,^51^ and adult substance users^52^ including those in OAT for OUD. Interventions targeting hopelessness have been shown to reduce SU.^32^ Similarly, youth living with parents who are prescribed opioids are at risk of exposure to opioids and potential future overdose.^20^ Our research was not able to identify interventions for these at-risk youth.

## Conclusion

Canadian youth continue to be a vulnerable group in the opioid overdose and health crisis. The evidence base for opioid use treatments among youth is lacking in North America and elsewhere in the world. The studies we found were not tailored to youth-specific socio-ecologies. The heterogeneity of youth experiences with opioids in North America may indicate that traditional approaches to treatment are not attuned to youth-specific intersecting vulnerabilities and contexts.

Within the context of current experimental interventions, we need a greater emphasis on understanding the factors that promote retention among young people, concerted efforts to include young women and gender diverse youth in treatments, programs that assess continued safer use within a paradigm of harm reduction, programs that focus on prevention of opioid use and address youth socio-economic positionalities, and treatment evaluation that seeks to “success” in these programs.

We argue researchers and healthcare professionals can cultivate youth-adult partnerships wherein they can systematically solicit youth feedback about interventions and treatments to improve treatment relevance and efficacy. There clearly needs to be a greater emphasis on understanding and identifying the factors that promote youth participation in opioid use programs and on authentic engagement with youth values and needs. ^8^

## Appendix

Search Terms:

young adult.mp. or Young Adult/

(youth* or adolescen* or teen*).ti,ab.

Drug Users.mp. or Drug Users/

(opiate* or opioid* or heroin*).ti,ab.

Analgesics, Opioid.mp. or exp Analgesics, Opioid/

exp Morphine Derivatives/

exp Prescription Drug Misuse/

exp Opioid-Related Disorders/

exp Street Drugs/

exp Drug Misuse/

(abus* or addict*).ti,ab.

drug inject*.ti,ab.

polydrug*.ti,ab.

Risk Factors/

Health Promotion/

health services/ or adolescent health services/ or community health services/ or emergency medical services/ or health services for transgender persons/ or health services, indigenous/ or mental health services/ or nursing care/ or nursing services/ or patient care/ or pharmaceutical services/ or preventive health services/ or rehabilitation/ or rural health services/ or social work/ or student health services/ or suburban health services/ or urban health services/ or women's health services/

"Early Intervention (Education)"/

preventive health services/ or "early intervention (education)"/ or early medical intervention/ or health education/ or needle-exchange programs/ or primary prevention/ or school health services/ or secondary prevention/ or tertiary prevention/

preventative health services.mp.

Harm Reduction/

Opiate Substitution Treatment/

Methadone/

Narcotic Antagonists/

exp Morphinans/

Substance Abuse Treatment Centers/

exp Psychotherapy/

(psychother* or psychosocial).ti,ab.

counseling.ti,ab.

opioid crisis.ti,ab.

opioid epidemic*.ti,ab.

adolescence.mp. or Adolescent/ or Adolescent.mp.

(codeine* or fentanyl or actiq or duragesic or fentora or hydrocodone or hysingla er or zohydro er or acetaminophen or lorcet or lortab or norco or vicodin or hydromorphone or dilaudid or exalgo or meperidine or demerol or methadone or dolophine or methadose or morphine or astramorph or avinza or kadian or ms contin or ora-morph sr or oxycodone or oxycotin or oxecta or roxicidone or percocet or endocet or roxicet or targiniq er).ti,ab.

4 or 5 or 6 or 7 or 8 or 9 or 10 or 11 or 12 or 13 or 14 or 32

15 or 16 or 17 or 18 or 19 or 20 or 21 or 22 or 23 or 24 or 25 or 26 or 27 or 28 or 29 or 30

((drug* or substance* or opioid*) adj2 (abuser* or addict* or user*)).ti,ab.

((recreational or casual) adj2 (drug* or substance* or opioid*) adj2 (abuser* or addict* or user*)).ti,ab.

1 or 2 or 31

3 or 35 or 36

37 and 38

39 and 33 and 34

limit 40 to (english language and humans and yr="2013 - 2021")
